# Driving forces of vancomycin-resistant E. faecium and E. faecalis blood-stream infections in children

**DOI:** 10.1186/2047-2994-3-29

**Published:** 2014-09-01

**Authors:** Maria Cecilia Di Pentima, Shannon Chan, Carol Briody, Michelle Power, Jobayer Hossain

**Affiliations:** 1 Infectious Diseases Division, Department of Pediatrics, Vanderbilt University, D-7235 Medical Center North, 1161 21st Avenue South, Nashville, Tennessee, USA; 2 Department of Pediatrics, Alfred I. duPont Hospital for Children, Wilmington, Delaware, USA; 3 Christiana Health Care System, Newark, USA; 4 Nemours Biomedical Research, Wilmington, Delaware, USA; 5 Department of Food and Resource Economics, University of Delaware, Newark, Delaware, USA

**Keywords:** *Enterococcus faecium*, *Enterococcus faecalis*, *Enterococcus* spp, Vancomycin, Bacteremia

## Abstract

**Background:**

Rates of invasive vancomycin-resistant *Enterococcus* (VRE) in the USA remains on the rise. Efforts to control vancomycin use and nosocomial transmission have had limited success in halting the spread of this pathogen. The role of antibiotic exposure remains a topic of controversy. We evaluated the association between emergence of VRE-blood-stream infections (BSI), aggregate and individual-patient vancomycin- exposure, and clonal transmission of VRE at an academic pediatric tertiary care hospital.

**Methods:**

*E. faecium* and *E. faecalis* isolates recovered from blood specimens from hospitalized children from 2003–2010 were retrieved from the microbiology database. Aggregate vancomycin use and individual-patient vancomycin exposure 6 months preceding each event of bacteremia were recorded. Pulse-field electrophoresis was performed on selected VRE isolates.

**Results:**

Of 151 episodes of *E. faecium* and *E. faecalis* BSI among hospitalized children <18 years of age, 9% (14) were due to VRE. Of these, 5 (36%) were due to nosocomial transmission. Aggregate (*r* .19, *P =* 0.3) and individual-patient vancomycin-exposure (*X*^*2*^ = .26; *P* = .87) were not associated with VRE-BSI. On bivariate analysis, OR for developing VRE-BSI among patients infected with clonal isolates was 36 (*P* < .0001). Infection control interventions, rather than antimicrobial stewardship interventions to decrease vancomycin use, proved to be effective in reducing the rates of VRE-BSI.

**Conclusions:**

In our experience, VRE-BSI was associated with nosocomial transmission and was independent of aggregate and individual-patient vancomycin-exposure. Molecular epidemiology is a crucial tool to differentiate the role of nosocomial transmission and antibiotic exposure in the emergence of invasive VRE infections among hospitalized children.

## Background

Over the past two decades, the emergence of vancomycin-resistant *Enterococcus* (VRE) as a nosocomial pathogen has been attributed to a complex interaction of epidemiologic forces including nosocomial transmission and antimicrobial pressure [1-3]. In particular, the use of selected antibiotics such as vancomycin, expanded-spectrum cephalosporins, and agents with potent anaerobic activity has been found to promote individual patients to gastrointestinal colonization with VRE [4]. Nevertheless, the role of antibiotic exposure in both colonization and infection remains controversial [3,5,6]. Efforts to control vancomycin use and nosocomial transmission have had limited success, and rates of invasive VRE in the USA remains on the rise [7]. Furthermore, mortality and hospital costs associated with VRE-BSI are significantly high, especially among immunocompromised patients [8].

We sought to evaluate the association between aggregate and individual-patient vancomycin-exposure, clonal transmission among hospitalized children and rates of VRE-BSI at a tertiary-care children hospital. To the best of our knowledge this is the first study addressing individual-patient and aggregate vancomycin exposure and nosocomial transmission using pulse-field electrophoresis to determine risk factors associated with VRE-BSI in pediatric patients.

## Methods

### Patients and settings

The study was performed at Alfred I. duPont Hospital for Children, Wilmington, DE, a tertiary, 180-bed, academic hospital affiliated with Thomas Jefferson University (Philadelphia, PA) [9,10]. From April 1st, 2003 to March 31st, 2010 clinical blood cultures reporting *E. faecalis and E. faecium* among hospitalized children were retrieved from the Microbiology Laboratory database. Records of the annual rates of solid organ transplants (SOT) (kidney, liver and heart transplants) and hematopoietic stem-cell transplant (HSCT) were obtained from the hospital executive statistical summary and the HSCT program.

### Laboratory methods

One isolate per patient per year was included in the analysis. *E. faecium* and *E. faecalis* isolates were identified following standard procedures at Alfred I. duPont Hospital microbiology laboratory. From April 1, 2003 to June 30, 2008, vancomycin susceptibility testing was performed by Etest® (AB Biodisk, BioMerieux, S.A., Marcy I’Etoile, France), and from July 1, 2008 to March 31, 2010, using a semi-automated system (MicroScan; Dade Behring, West Sacramento, CA).

Pulsed-field electrophoresis (PFGE) for selected isolates was performed at a reference laboratory (ARUP Laboratories, Salt Lake City, UT) [11]. Isolates were classified based on published criteria as indistinguishable, closely-related, and unrelated [12].

### Infection control practices

Active surveillance for hospital-associated BSI was routinely performed by the infection control department. In October 2007, an increased number of VRE-BSI was noted among transplant patients hospitalized in the Pediatric Intensive Care Unit. Surveillance stool cultures was implemented for patients admitted to the intensive case care, HSCT and oncology units upon admission and weekly until discharge in October 2008.

### Measures of antibiotic use

Aggregate vancomycin use April 1st, 2003 to March 31st, 2010 were captured by number of doses administered (DA) to each unique patient and normalized per 1000 patient-days to control for differences in the annual hospital census [9]. Vancomycin therapy, expressed as the number of DA and days of therapy (DoT), for each individual patient during the 6 months preceding each event of *E. faecalis and E. faecium* BSI was recorded.

### Analysis

Temporal trends of aggregate vancomycin use per 1000 patient-days were analyzed using a *X*^2^ test trend for proportions. Spearman’s correlation coefficient was used to evaluate the association between aggregate vancomycin use and VRE BSI; and rates of SOT and HSCT and rates of VRE BSI. Chi-square was used to evaluate the distribution of individual-patient vancomycin-exposure and VRE-BSI. Logistic regression analysis was used to examine the association of VRE-BSI and clonal transmission, while controlling for age and gender. All tests were two-tailed with a .05 as the level of significance. Analyses were performed using IBM-SPSS Software (Version 20.0, IBM Corp) and Statistical software R (Version 2.10.2).

The Institutional Review Board of Nemours approved this study.

## Results

### *E. faecium* and *E. faecalis* blood-stream infections

From April 1st, 2003 to March 31st, 2010, we identified 151 episodes of *E. faecium* and *E. faecalis* bacteremia in 101 pediatric patients (46 females; 55 males). Demographic characteristics are depicted in Table 1. The majority (137 of 151; 88%) of *Enterococci* BSI were due to vancomycin-susceptible *E. faecium* and *E. faecalis* (VSE). Of these, *E. faecalis* and *E. faecium* were responsible for 104 (76%) and 33 (24%) episodes of BSI, respectively. Infants less than one year of age represented 42% (57 of 137) of all episodes of VSE-BSI.

**Table 1:** **Demographic characteristics of *Enterococcus faecium *** **and *Enterococcus faecalis *** **blood-stream infections at Alfred I. duPont Hospital for Children, 2003-2010**

**Characteristics**	**Total N = 151**	**N = 14 (%)**		**Vancomycin-Susceptible** ***Enterococcus *** **spp N = 137 (%)**	
		***E. faecalis***	***E. faecium***	***E. faecalis***	***E. faecium***
		**N = 3 (21)**	**N = 11 (79)**	**N = 104 (76)**	**N = 33 (24)**
Median age in years (range)	1 (0-20)	3 (0 - 4)	4 (0 - 16)	1 (0-20)	1 (0 - 19)
Male, No (%)	82 (54)	1 (1)	4 (5)	58 (71)	19 (23)
CR-BSI^*^, No (%)	101 (68)	3 (3)	6 (6)	66 (65)	26 (26)

*Catheter-related blood-stream infection.

Fourteen (14 of 151, 9.2%) episodes of bacteremia were associated with VRE in 11 individual patients (Table 1). One small-bowel transplant patient had three episodes of VRE blood-stream infections over a 4-year period. The first and third episode (2004 and 2007), were caused by *E. faecium*, while the second episode was caused by *E. faecalis* (2005). A renal transplant patient had two episodes of bacteremia 10 months apart. In the unadjusted model, girls were more likely to develop VRE-BSI (OR = 1.45; *P* = .63) (Table 1). When compared with younger and older children, those between 2 and 6 years of age were at higher risk of developing VRE blood-stream infection (OR = 3.8; CI = 1-14; *P* = .055).

**Figure 1:**
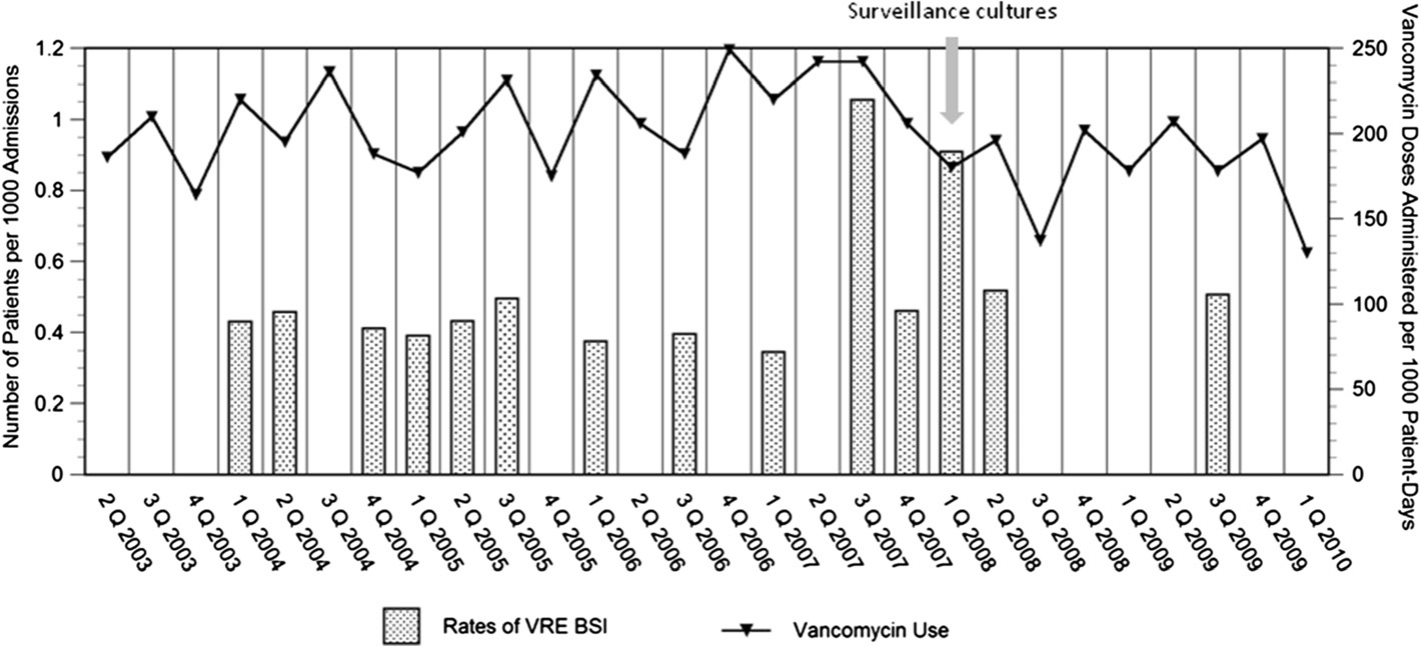
**Rates of vancomycin-resistant of *E. faecalis *** **and *E. faecium *** **blood-stream infections and aggregate vancomycin use (expressed as the number of doses administered per 1000 patient-days), Alfred I. duPont Hospital for Children, April 1, 2003- March 31st, 2010.**

VRE-BSI were sporadic between April, 2003 and July, 2008 (Figure 1). Rates of VRE-BSI peaked during the last two quarters of 2007, and declined after strict infection control measures and surveillance were implemented (Figure 1). Of the 5 patients with VRE-BSI between September, 2007 and April, 2008, 4 were associated with vancomycin-resistant *E. faecium.* Three had undergone HSCT (two infants received cord-blood transplantation for congenital hemophagocytic syndrome, and the third patient underwent autologous peripheral blood stem cell for metastatic retinoblastoma) and one patient had undergone liver and renal transplantation. One additional liver transplant patient developed vancomycin-resistant *E. faecalis* bacteremia. The overall mortality among transplant patients was 50%; however, the case fatality rate among HSCT patients was 67%, despite prompt and appropriate antibiotic therapy. No deaths occurred among SOT patients. We did not find a statistically significant correlation between rates of HSCT (*r =* .44, *P* = .3) and SOT (*r =* .22, *P* = .6), and rates of VRE-BSI.

Between September, 2007 and April, 2008, 17 isolates (5 from blood and 12 from stool) were submitted for PFGE to determine clonality. Of these, 5 isolates were indistinguishable (29%; 5/17). Four of these were among the *E. faecium* isolates recovered from patients with blood-stream infections. Three additional *E. faecium* isolates were closely-related and 5 other strains were unrelated. On bivariate analysis, the odds ratio for developing VRE blood-stream infection for patient with clonal transmission was 36 (CI = 7-167; *P* < .0001). After controlling for age and gender, this odds ratio was 41.5 (CI = 7-250; *P* < 0.001).

A single patient developed VRE-BSI between April, 2008 and March, 2010.

### Infection control interventions

Contact precautions were implemented in all newly admitted patients pending the results of the stool surveillance cultures. Patients with negative results were removed from contact isolation, but weekly screening continued during the length of their hospitalization. Infection control policies included: 1. Staff were required to wear gowns and gloves, and perform hand hygiene before and after entering a room. 2. Patients remained in their room without access to common places. 3. Patients traveling to another part of the hospital used gown and gloves. 4. ‘Terminal cleaning’ was performed after patient’s discharge. Additional interventions included: 1. “Bare Below the Elbows” rule, eliminating white coats, jackets, ties and jewelry. 2. Dedicated equipment and supplies to isolation rooms. 3. Reinforcement of housecleaning activities: spot-cleaning high touch surfaces with CaviWipes and de-cluttering of the rooms. 4. Limit toys available in the rooms. Skin de-colonization of colonized patients was implemented using Sage 2% CHG cloths.

### Vancomycin exposure

Following the implementation of an antimicrobial stewardship program in 2004, the use of vancomycin decreased from 378 DA/1000 patient-days per year in 2003–2004 to 173 DA/1000 patient-days per year in 2009–2010 (*P* = <0.001) [10,13]. Vancomycin use was not associated with increased use of other anti-staphylococcal antibiotics as previously reported [10,13]. Aggregate use of vancomycin did not correlate with rates of VRE-BSI (*r =* .19, *P =* 0.3).

Of the 151 episodes of *E. faecalis and E. faecium* BSI, 105 (69.5%) were documented in patients who received at least one dose of vancomycin within the previous 6 months. Individual-patient vancomycin-exposure was not associated with an increased risk for VRE-BSI (*X*^*2*^ *=* .26, *P* = .87). On average, previous vancomycin-exposure among patients with VSE*-*BSI was 32 DA (range, 1–196) and 14 DoT (range, 1–68). Patients who developed VRE-BSI received 24 DA (range, 2–68) and 13 DoT (range, 1–196) of vancomycin prior to their events of bacteremia.

## Discussion

*Enterococcus* spp. bacteremia is common in children with intraluminal pathology and at higher risk for gastrointestinal translocation. In our experience, the majority of *Enterococcus* spp BSI were noted in infants and the majority of these were associated with VSE. Conversely, VRE-BSI was mainly seen in immunocompromised children, the majority, undergoing transplantation. Since the first hospital-acquired VRE infection was described in France, rates of VRE infections remain on the rise [2,7]. Selective antibiotic pressure and environmental contamination are well described risk factors associated with acquisition of VRE [14]. Antibiotics without activity against VRE achieving high concentrations in the gastrointestinal tract have been shown in humans and animal models to promote VRE colonization [6]. In our experience, nosocomial transmission was associated with emergence of VRE-BSI among the most vulnerable patient populations. Nosocomial transmission was rapidly controlled by implementing strict infection control interventions. The implementation of an integrated antimicrobial stewardship program led to an overall reduction of vancomycin and broad-spectrum antibiotic use [9]. During the 7 years of this study, aggregate and individual-patient vancomycin-exposure were not found to be associated with VRE-BSI among hospitalized children. Similarly, Duchon and colleagues reported that neonates colonized and/or infected with VRE had less days of vancomycin therapy when compared with infants hospitalized in the same intensive care unit who were not colonized with VRE [2].

## Conclusions

Controlling the forces driving the emergence of invasive VRE infection requires an interdisciplinary approach and commitment. Furthermore, studies assessing the impact of antimicrobial stewardship interventions and emergence of antibiotic resistance could be bias by unrecognized nosocomial transmission of multidrug-resistant organisms. Molecular epidemiology is a powerful tool to discern between antibiotic exposure and hospital transmission as potential sources of VRE-BSI. In our experience, close vigilance of environmental contamination and enforcement of infection prevention and antibiotic policies proved efficient to control nosocomial transmission and reduce the rates of VRE-BSI in subsequent years. These strategies are warranted to reduce mortality and costs associated with VRE-BSI among the most vulnerable hospitalized pediatric patients.

## Competing interests

All authors report no conflicts of interest relevant to this article.

## Authors’ contributions

MCDP, SC, CB, and MP made substantial contributions to conception and design, and acquisition of data. JH made a substantial contribution to the statistical analysis and interpretation of data. MCDP drafted the manuscript, and all authors gave final approval of the version to be published.
